# Clinical Benefits of the Introduction of the ERAS Protocol in Thyroid Surgery: A Propensity Score Matching Analysis

**DOI:** 10.3390/jcm15114106

**Published:** 2026-05-26

**Authors:** Giacomo Di Filippo, Simona Mastrangeli, Giulia Gobbo, Giovanni Lazzari, Eleonora Morelli, Dorin Serbusca, Marco Mazzola, Federica Cantaluppi, Beatrice Milan, Enrico Polati, Katia Donadello, Andrea Ruzzenente

**Affiliations:** 1 Endocrine Surgery Unit, Department of Surgery and Oncology, Verona University Hospital, 37134 Verona, Italy; 2 Anaesthesia and Intensive Care Unit B, University Hospital Integrated Trust of Verona, 37134 Verona, Italy; 3 Otolaryngology-Head and Neck Surgery Department, University of Verona, 37134 Verona, Italy; 4 Department of Surgery, Anaesthesia and Intensive Care Unit B, Dentistry, Paediatrics and Gynaecology, University of Verona, 37134 Verona, Italy; 5 Department of Surgery, University of Verona, 37134 Verona, Italy

**Keywords:** Enhanced Recovery After Surgery (ERAS), thyroidectomy, lobectomy, length of stay, hypocalcemia

## Abstract

**Background:** The ERAS (Enhanced Recovery After Surgery) protocol is a multidisciplinary perioperative care pathway aimed at improving postoperative recovery. Few studies have investigated their effectiveness in thyroid surgery. The aim of this study was to evaluate the potential clinical benefits of a structured ERAS protocol applied to thyroid surgery in a tertiary referral center. **Methods:** We conducted a single-center retrospective study on consecutive series of patients undergoing thyroid surgery for benign or malignant disease before (January 2018–December 2022) and after (May 2024–July 2024) the introduction of the ERAS protocol. A propensity score matching (PSM) analysis was used, matching the groups for age, gender, BMI and type of surgery. **Results:** After PSM, 64 matched patient pairs were analyzed. ERAS patients showed a significant reduction in length of hospital stay (*p* < 0.001), postoperative nausea and vomiting (3.1% vs. 12.5%, *p* = 0.04), and pain with an NRS > 5 12 h after surgery (9.4% vs. 26.6%, *p* = 0.01). Patients in the ERAS group showed significantly higher calcium levels at 6 h (9.09 vs. 8.7 mg/dL, *p* < 0.001) and 24 h (8.8 vs. 8.6 mg/dL, *p* = 0.008) postoperatively, with a reduction in the need for intravenous calcium therapy (0% vs. 12.5%, *p* = 0.003). **Conclusions:** This study showed significant clinical benefits and reduced length of stay were achieved in thyroid surgery patients after the implementation of a structured ERAS protocol with potential implications for healthcare cost reduction.

## 1. Introduction

The Enhanced Recovery After Surgery (ERAS) protocols are evidence-based, multimodal, and multidisciplinary programs implemented in the perioperative setting to attenuate the patient’s surgical stress response and promote a rapid return to baseline functional status.

ERAS was first conceptualized by Henrik Kehlet in the context of colorectal surgery [1], where it was initially developed and implemented. Over time, ERAS pathways in colorectal surgery have been widely adopted, and since 2005, the ERAS Society has recommended the routine use of a standardized ERAS protocol to improve the care of patients undergoing major colorectal surgery [2].

Meanwhile, the ERAS principles have been extended to multiple surgical specialties, leading to the development of different pathways tailored to the specific characteristics of each surgical field [3,4,5,6]. Previous evidence across multiple surgical subspecialties has demonstrated that the systematic implementation of these specialty-adapted ERAS pathways is associated with reduced hospital length of stay and healthcare costs, without an increase in postoperative complications or readmission rates [7,8,9,10].

In thyroid surgery, the application of ERAS pathways has also been increasingly explored, although the literature is still limited, with several studies reporting benefits across different perioperative domains [11]. For instance, Chen et al. implemented an ERAS protocol for transoral robotic thyroidectomy (TORT) that incorporated nebulized dexamethasone to reduce postoperative sore throat, and structured early mobilization initiated as early as two hours after surgery [12]. Similarly, Yip et al. developed a pathway focused on minimizing opioid use through total intravenous anesthesia (TIVA) with propofol, preoperative gabapentin, and intraoperative cervical blocks, achieving an approximately 70% reduction in in-hospital opioid use [13]. More recently, Machado et al. focused on multimodal analgesia and prophylaxis strategies, including pre-incision superficial cervical plexus blocks, preoperative celecoxib administration, and risk-adapted prevention of postoperative nausea and vomiting, thereby facilitating safe same-day discharge [14].

Despite the reported benefits and unlike colorectal surgery and other specialties, a standardized ERAS protocol for thyroid surgery, formally endorsed by the ERAS Society, is not currently available. This inevitably affects both clinical practice and research, hindering consistent implementation across institutions and reducing comparability between studies.

Within this context, we developed a structured institutional ERAS pathway for patients undergoing thyroid surgery at our Institution, a tertiary referral center for endocrine surgery. The aim of the present study was to compare surgical outcomes in patients undergoing thyroidectomy before and after implementation of this pathway.

## 2. Materials and Methods

### 2.1. Study Design and Setting

The present study is a single-center retrospective study conducted at a tertiary referral center for endocrine surgery (Verona University Hospital).

The study was approved by the Ethics Committee of Veneto South West Area. All procedures were conducted in accordance with the principles of the Declaration of Helsinki.

### 2.2. Eras Protocol

A multidisciplinary team including surgeons, anesthesiologists, nurses, rehabilitation specialists, and dietitians was involved in the development of a structured ERAS protocol for thyroid surgery, with the aim of improving patient outcomes. The protocol components were defined based on evidence-based practice and available literature.

The ERAS protocol implemented for the purposes of the present study consists of three phases: preoperative, intraoperative, and postoperative.

In the preoperative phase, patients receive detailed information through educational brochures describing the protocol and emphasizing the importance of adherence to perioperative recommendations. Patients are encouraged to follow a balanced diet (based on the principles of the Mediterranean Diet), engage in daily physical activity (at least a 30-min walk), and discontinue smoking and alcohol consumption. In selected cases, such as obese patients, sarcopenic patients, or those with reduced cervical mobility, dietary and physiatric consultations are provided. In patients with vitamin D deficiency, supplementation with cholecalciferol (50,000 IU weekly for four weeks) is prescribed.

On the day before surgery, patients are allowed to eat until midnight, while clear fluids are permitted up to three hours before the procedure. Thromboprophylaxis consists of elastic compression stockings for all patients, with the addition of subcutaneous low molecular weight heparin in selected cases at high risk for venous thromboembolism. Antibiotic prophylaxis (cefazolin 2 g) is selectively administered to patients at high risk for infection (e.g., obesity, alcohol abuse, non-independent functional status, diabetes mellitus, immunosuppression, operative time > 3 h, or airway injury).

Preemptive pharmacological management includes paracetamol 1 g, ketorolac 30 mg, pantoprazole 40 mg, and midazolam at the anesthesiologist’s discretion, along with dexamethasone 4 mg, with or without ondansetron according to the Apfel score for postoperative nausea and vomiting (PONV) prophylaxis. Total intravenous anesthesia (TIVA) with propofol, with minimal opioid use, is the preferred anesthetic technique.

In the intraoperative phase a minimally invasive approach (i.e., minicervicotomy, defined as an incision < 4 cm) is performed whenever feasible. Local infiltration with ropivacaine (75 mg) is performed. Intermittent intraoperative neuromonitoring and advanced hemostatic devices (e.g., advanced bipolar hemostats) are routinely used. At the end of the procedure, hemostasis is carefully reassessed under positive end-expiratory pressure (8–12 cm H_2_O) and in the Trendelenburg position to optimize bleeding control.

Urinary catheterization is not routinely performed, except in procedures expected to last more than 4 h. Surgical drains are not routinely placed and are used at the discretion of the lead surgeon; when used, they are removed after 24 h in the absence of bleeding complications. Active intraoperative warming using a patient-warming blanket is applied to prevent hypothermia.

In the postoperative phase, analgesia consists of paracetamol 1 g three times daily, with rescue analgesia using ketoprofen 160 mg or tramadol within the first 24 h. Thereafter, oral analgesia with paracetamol 1 g or ibuprofen 400 mg is administered as needed. In cases of nausea or vomiting, ondansetron 4 mg is provided.

Patients are encouraged to resume oral intake and mobilization six hours after surgery. Discharge criteria include stable vital signs, adequate oral intake, independent mobilization, and absence of cervical bleeding.

**Figure 1:**
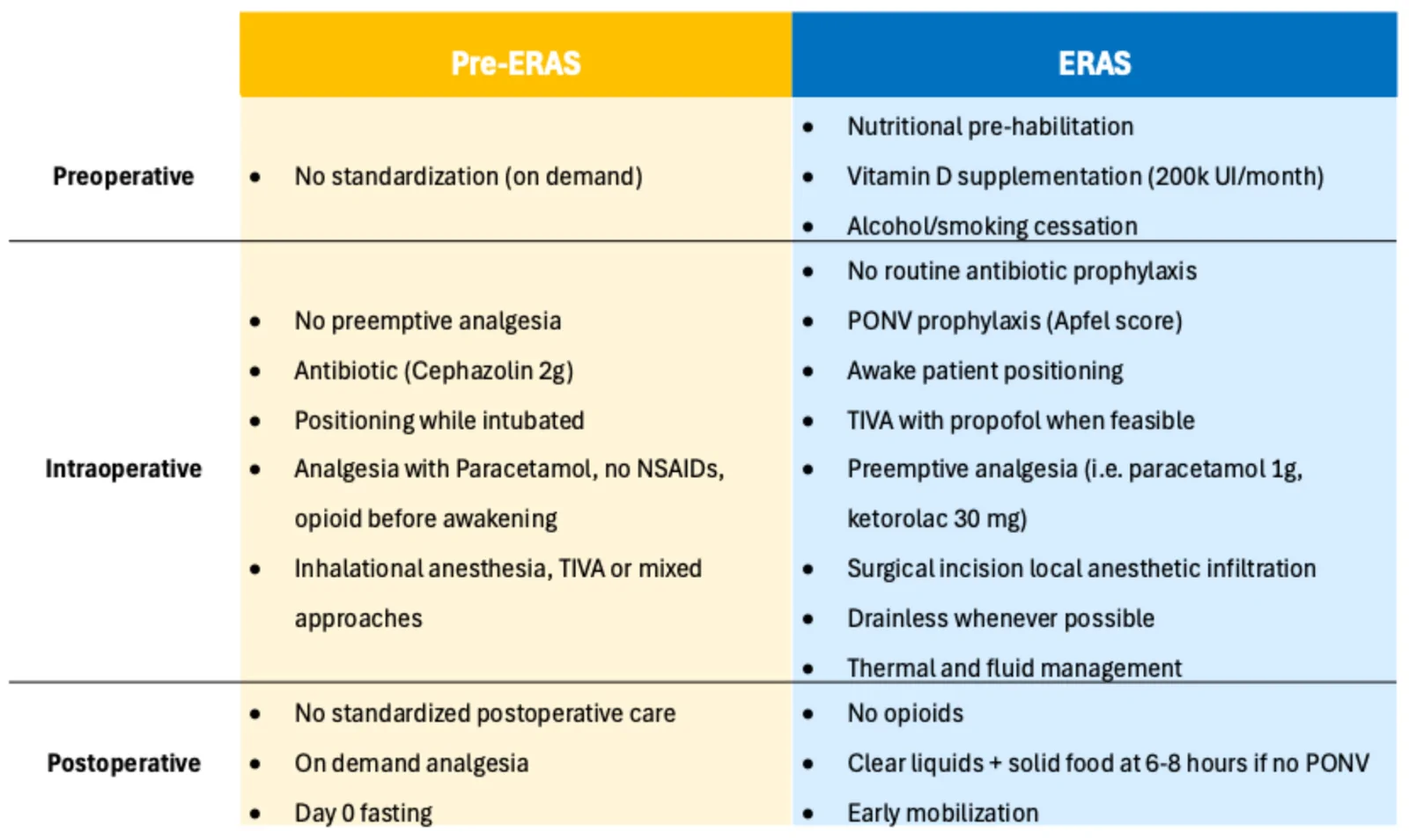
Differences between ERAS protocol and previous standard care. PONV: Postoperative Nausea and Vomiting; TIVA: Total Intravenous Anesthesia; NSAIDs: Non-Steroidal Anti-Inflammatory Drugs.

The described ERAS protocol has been implemented for all patients undergoing thyroid surgery from May 2024 onward. A summary of the differences between the ERAS protocol and previous standard care is provided in Figure 1.

### 2.3. Patients

**Figure 2:**
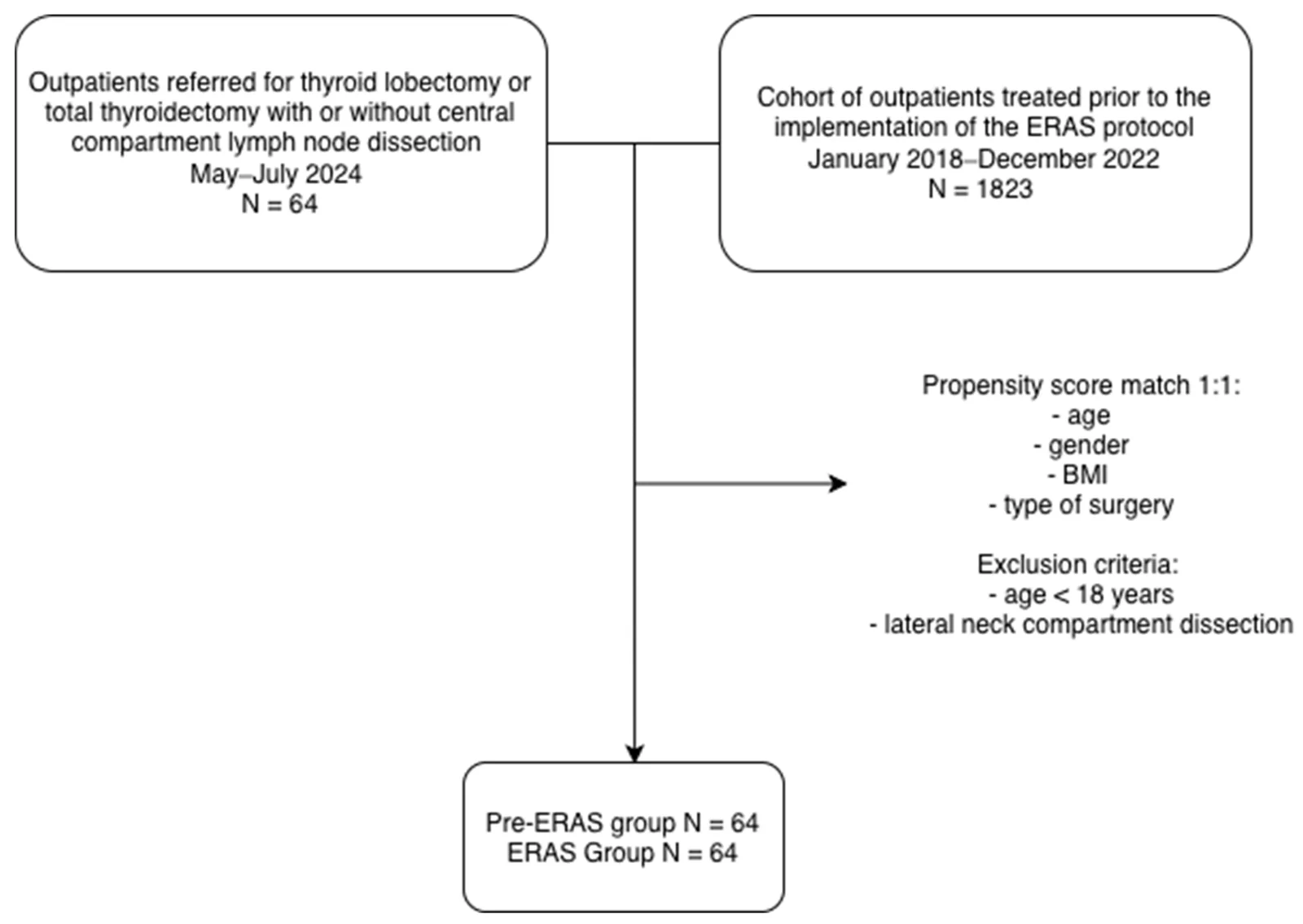
Flowchart describing the study design.

The study population included all consecutive adult patients undergoing thyroid lobectomy or total thyroidectomy, with or without central compartment lymph node dissection, between May 2024 and July 2024 (ERAS group). This cohort was compared with a historical cohort of patients treated prior to the implementation of the ERAS protocol, between January 2018 and December 2022 (Pre–ERAS group). Inclusion criteria were: age ≥ 18 years, thyroid lobectomy or total thyroidectomy with or without central compartment lymph node dissection. The exclusion criteria were as follows: age < 18 years, inability to comply with the ERAS protocol during the preoperative period, coexistent psychiatric disease, hyper-hypoparathyroidism or calcium disorders and lateral neck compartment dissection (Figure 2). Written informed consent for the use of clinical data for research purposes was obtained from all patients included in the study.

Postoperative pain was assessed using the Numerical Rating Scale (NRS) [15], in accordance with our institutional protocol.

Postoperative hypocalcemia was defined as a total serum calcium level below the lower limit of the institutional laboratory reference range (8.5–10 mg/dL).

### 2.4. Statistical Analysis

A 1:1 propensity score matching analysis was performed to minimize selection bias. Matching variables included age, gender, body mass index (BMI), type of surgery, and preoperative vitamin D supplementation. This approach ensured comparable groups between ERAS and Pre-ERAS cohorts. Categorical variables were expressed as absolute frequencies and percentages, while continuous variables were reported as median values with interquartile ranges (IQR). Comparisons between groups were performed using the chi-square test for categorical variables and the Mann–Whitney U test for continuous variables. A *p* value < 0.05 was considered statistically significant. Statistical analysis was performed using SPSS (Statistical Package for the Social Sciences, IBM SPSS Statistics for Windows, Version 25.0. Armonk, NY, USA: IBM Corp.).

## 3. Results

The study included 64 consecutive patients who underwent thyroid surgery between 1 May 2024 and 31 July 2024 following the implementation of the ERAS protocol (ERAS group). These patients were matched in a 1:1 ratio using propensity score matching with a cohort of 1823 consecutive patients treated between 1 January 2018 and 31 December 2022, resulting in 64 matched patients (Pre–ERAS group).

The final matched cohort comprised 128 patients, whose baseline characteristics are summarized in Table 1, while surgical results are summarized in Table 2.

**Figure 3:**
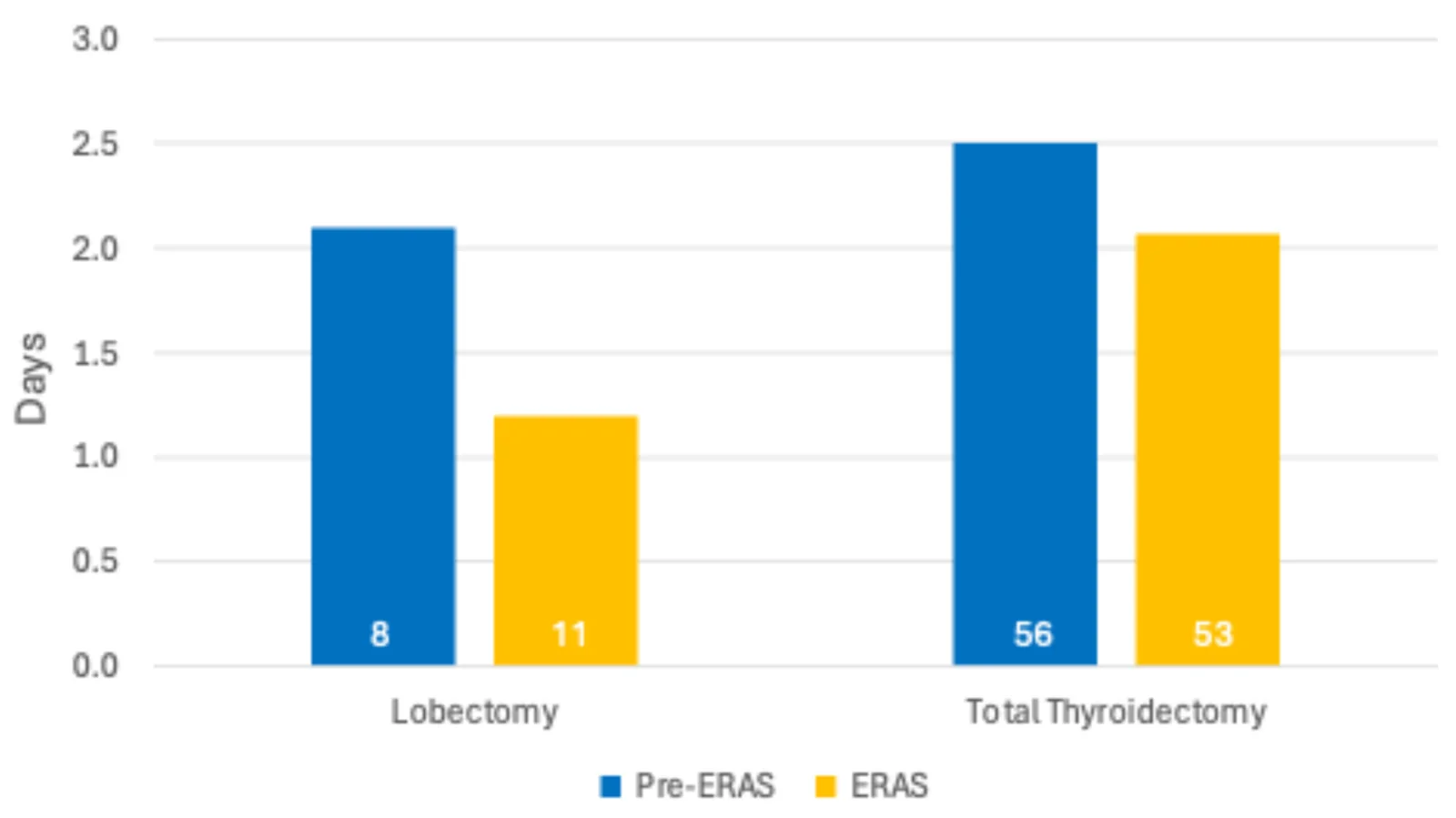
Mean length of stay according to the type of surgery in Pre-ERAS vs. ERAS group.

**Figure 4:**
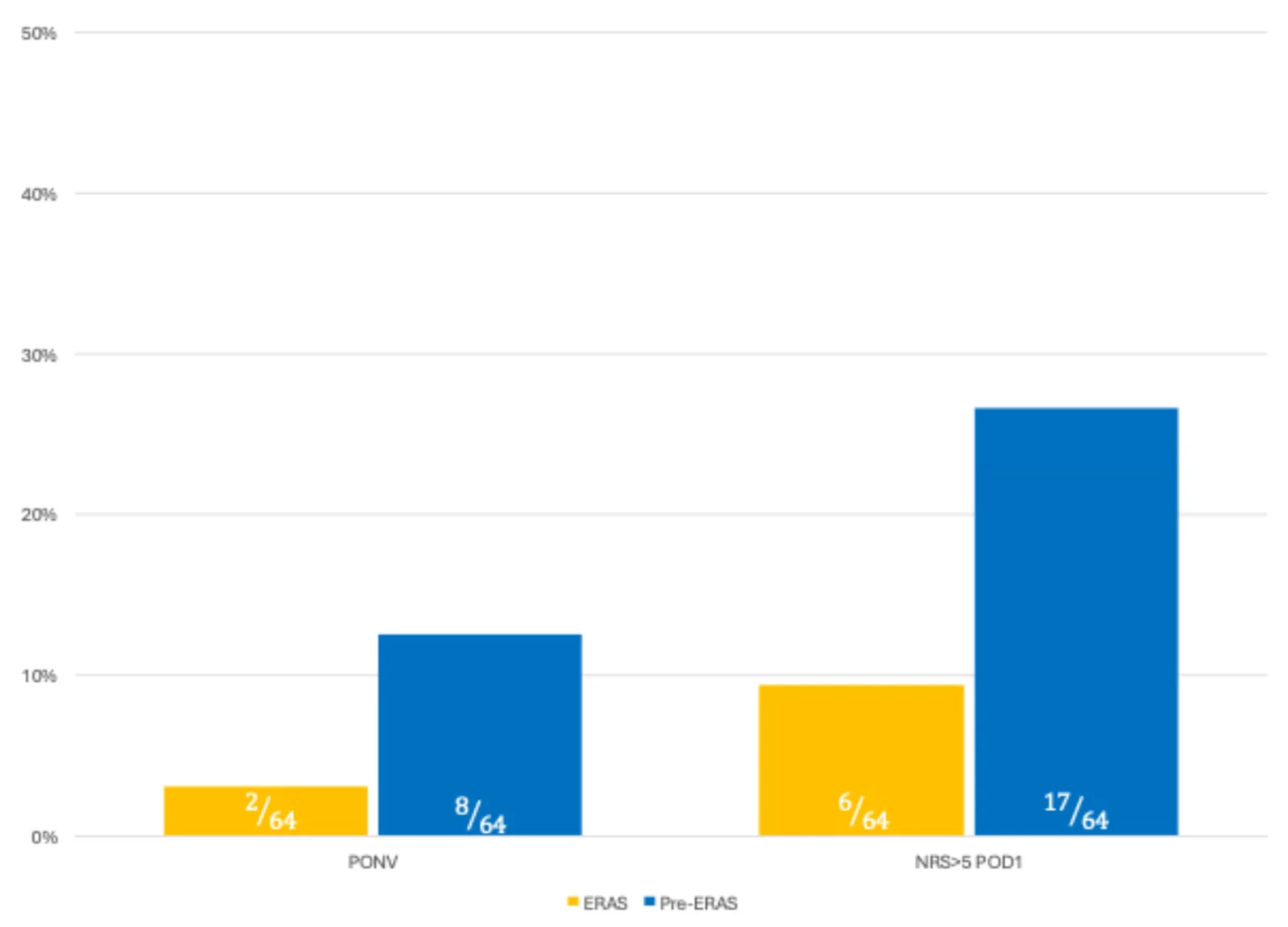
PONV and NRS > 5 in POD1 in Pre-ERAS vs. ERAS group. PONV: Post Operative Nausea and Vomiting; POD1: Post Operative Day 1.

**Figure 5:**
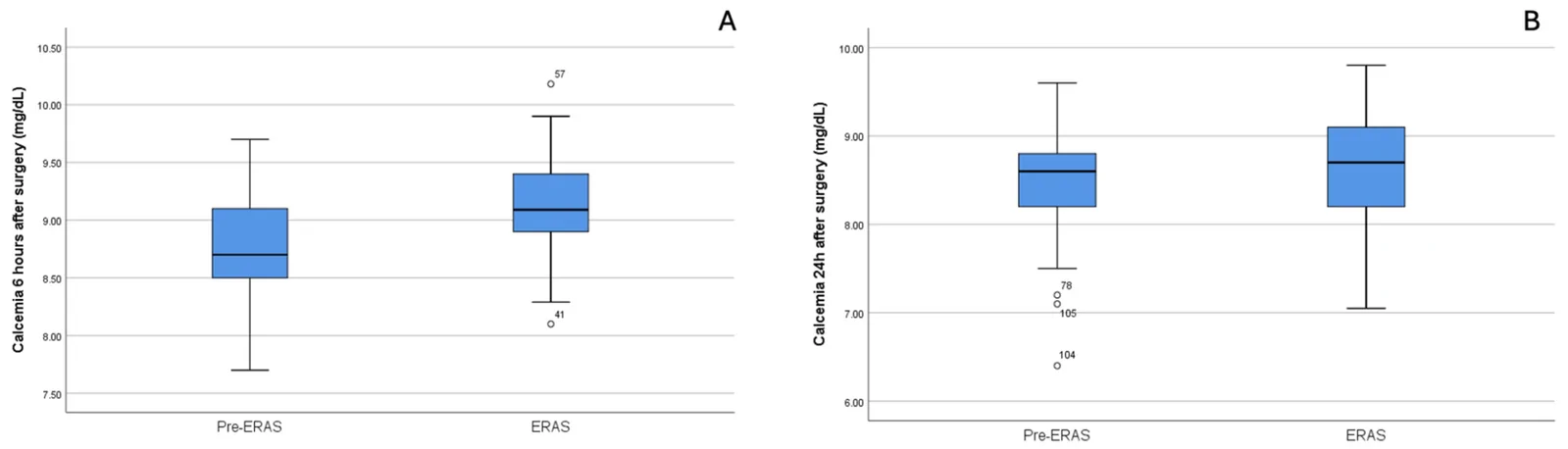
Boxplots illustrating serum calcium levels measured at 6 (**A**) and 24 h (**B**) after surgery.

Compared to the Pre-ERAS group, ERAS group patients had a significantly shorter mean length of hospital stay (2.41 vs. 1.95 days; *p* < 0.001), especially those undergoing lobectomy (Figure 3), and a significantly lower rate of postoperative nausea and vomiting (PONV) (3.1% vs. 12.5%, *p* = 0.04) and of NRS > 5 pain during the first 12 h after surgery (9.4% vs. 26.6%, *p* = 0.01) (Figure 4). Additionally, patients in the ERAS group showed significantly higher calcium levels at 6 (9.09 vs. 8.7 mg/dL, *p* < 0.001) and 24 h (8.8 vs. 8.6 mg/dL, *p* = 0.008) (Figure 5) postoperatively, with a lower need for intravenous calcium gluconate therapy for symptomatic hypocalcemia (0% vs. 12.5%, *p* = 0.003).

No significant differences were observed between the two groups in terms of operative time or other postoperative complications, including hematomas, recurrent laryngeal nerve palsy, and surgical site infections.

## 4. Discussion

The present study evaluated the effects of implementing a structured evidence-based ERAS protocol in thyroid surgery to optimize patients’ perioperative course through multidisciplinary management in an Italian tertiary referral center for endocrine surgery.

Despite the increasing adoption of ERAS pathways across multiple surgical specialties, evidence on their implementation in thyroid surgery remains scarce. A possible reason for this may be the perceived low morbidity and fast postoperative recovery following this type of surgery. Within this context, our study aimed to contribute additional evidence supporting the feasibility and clinical benefits of a structured ERAS protocol in thyroid surgery.

Within our population, the implementation of a structured ERAS protocol for thyroid surgery resulted in higher postoperative calcium levels, lower incidence of symptomatic hypocalcemia, improved pain and PONV control, as well as reduced length of stay (LOS).

In our analysis, patients managed within the ERAS clinical pathway demonstrated higher postoperative calcium levels at both 6 and 24 h. Moreover, they experienced a lower incidence of symptomatic hypocalcemia and required less intravenous calcium gluconate supplementation. This effect may be attributable, at least in part, to the systematic preoperative correction of vitamin D deficiency implemented within the ERAS protocol, and is consistent with previous literature aligning with recommendations from the American Thyroid Association regarding the prevention and management of hypoparathyroidism [16]. The implementation of standardized preoperative vitamin D or calcium supplementation protocols within ERAS clinical pathways has been shown to reduce postoperative hypocalcemic events [17,18]. Mehreen et al. reported that the risk of developing postoperative hypocalcemia was 3.1 times higher in the placebo group compared to the supplementation group [19]. Additionally, a meta-analysis by Canali et al. highlights that vitamin D administration significantly reduces the risk of postoperative hypocalcemia and symptomatic hypocalcemia in patients undergoing total thyroidectomy [20]. In light of these results, we advocate for the preoperative correction of vitamin D deficiency, when clinically feasible, in order to reduce the risk of symptomatic postoperative hypocalcemia.

Despite the relatively short data collection period for the ERAS cohort, a higher number of seroma formations and surgical site infections was observed compared to the pre-ERAS group. However, these differences did not reach statistical significance. Given the rarity of these complications and the numerical imbalance between the pre-ERAS and ERAS cohorts, the non-statistically significant higher number of seromas and surgical site infections observed in the ERAS group may simply reflect random variation related to the propensity score-matched sample selection rather than a true increase in complication rates. To further clarify these findings, larger studies with bigger samples and longer data collection periods would be needed.

In our analysis, the implementation of a structured ERAS protocol resulted in a significant improvement in post-operative pain control, as measured by a lower incidence of NRS > 5 on post-operative day 1, and in a reduced incidence of PONV (3.1% vs. 12.5%) compared to the pre-ERAS group. These results were achieved through the adoption of a multimodal, opioid-sparing analgesic strategy. While our previous practice was largely based on paracetamol combined with opioids due to concerns regarding NSAID-related bleeding, the ERAS protocol introduced a structured regimen including paracetamol, ketorolac, and loco-regional techniques (e.g., bilateral superficial cervical plexus block or wound infiltration). This approach enabled effective pain control while limiting opioid use to rescue therapy, thereby reducing overall opioid exposure. Notably, in our study population, these outcomes were achieved without the need for more complex agents such as ketamine or gabapentinoids. Furthermore, although NSAIDs have historically been avoided in thyroid surgery due to bleeding concerns [21,22], emerging evidence suggests that their judicious use does not significantly increase clinically relevant hemorrhagic complications, supporting their inclusion in ERAS pathways [23,24,25].

Our results are in line with previous research showing that the implementation of ERAS protocols in thyroid surgery significantly reduces postoperative pain and opioid use, while maintaining effective control of PONV [14]. For instance, Yip et al. reported that a structured ERAS pathway including the preoperative use of paracetamol and gabapentin alongside local anesthetic infiltration was associated with a 71.8% reduction in mean oral morphine equivalents, along with decreased need for ondansetron and scopolamine [13].

From a pathophysiological perspective, multimodal analgesia targets multiple steps of the nociceptive pathway, acting at both peripheral and central levels. By combining systemic non-opioid agents with regional techniques that reduce afferent nociceptive input, this strategy likely attenuates both inflammatory nociceptor activation and central sensitization, resulting in improved early postoperative pain control.

Importantly, the reduction in opioid use observed in our study likely contributed to the significant decrease in PONV rates within the ERAS group. Thyroid surgery is associated with a high baseline risk of PONV [26,27], making this outcome particularly relevant. In our protocol, antiemetic prophylaxis was guided by Apfel risk stratification [28], allowing tailoring of therapy according to individual risk. Antiemetic prophylaxis included the routine use of preoperative dexamethasone, which has been consistently shown to reduce PONV and enhance early recovery [29], in combination with ondansetron, when clinically indicated.

The observed improvement in PONV rates therefore reflects the effect of a multifactorial strategy, combining opioid minimization through TIVA-based anesthesia and multimodal analgesia with risk-adapted pharmacological prophylaxis.

In our study population, patients managed within the ERAS pathway experienced a shorter mean LOS than those in the pre-ERAS group, with the greatest reduction observed in patients undergoing thyroid lobectomy, without increasing the risk of complications.

Previous research has consistently demonstrated that the implementation of ERAS protocols in thyroid surgery is associated with a reduction in LOS [3,30,31,32]. Furthermore, a recent meta-analysis conducted by Chorath et al. showed an overall mean reduction of 0.64 days in hospital stay among patients managed within ERAS protocols, without compromising patient safety [33].

While some authors [34,35,36] have advocated for same-day discharge following thyroid surgery in selected patients, others caution against its adoption due to the risk of hard-to-predict, potentially life-threatening, postoperative complications [37,38,39]. In a European multicenter analysis, Canu et al. reported that most postoperative hematomas requiring surgical revision developed within the first 24 h after thyroid surgery, 74.28% of which developed within the first 6 h [40]. Accordingly, although the implementation of ERAS protocols may contribute to a meaningful reduction in LOS, as seen in our study, this effect should not be pursued indiscriminately, especially when trying to achieve same-day discharge in selected patients. Rather, we believe that early discharge strategies should be balanced against the need for appropriate postoperative surveillance to preserve patient safety.

We believe this study has significant strengths. The implementation of a standardized ERAS protocol in a high-volume tertiary referral center ensured close monitoring of protocol adherence. In addition, propensity score matching improved comparability between the ERAS and pre-ERAS groups, reducing selection bias and strengthening the validity of our findings. Finally, the multidisciplinary design of the ERAS pathway supports the practical applicability and reproducibility of our results in routine clinical practice.

Nonetheless, some limitations should be taken into account when interpreting our study results. First, the single-center design and retrospective nature may limit the generalizability of our findings. Although the use of propensity score matching helped create well-balanced groups and reduce selection bias, residual confounding cannot be entirely excluded. In addition, the implementation of the ERAS pathway involved multiple concurrent perioperative modifications, several of which may have independently influenced the postoperative outcomes of interest. At the same time, the fact that the same professionals were involved in both protocol development and patient management may have introduced performance bias. Furthermore, unmeasured changes in perioperative care unrelated to the ERAS protocol may have occurred during the 5-year pre-ERAS period, potentially introducing confounding related to evolving clinical practice over time. Lastly, the relatively small sample size may have limited our ability to detect differences in less frequent outcomes.

## 5. Conclusions

The introduction of a structured ERAS protocol in thyroid surgery in our center resulted in significant clinical benefits, including reduced postoperative pain, nausea, and vomiting, as well as improved calcium management. Additionally, the standardized perioperative care implemented contributed to a faster recovery and earlier discharge of thyroid surgery patients, which could potentially reduce healthcare costs. However, these findings should be interpreted with caution in light of the study’s inherent limitations, given the single-center, retrospective design, the potential for residual and temporal confounding, and the multifactorial nature of the ERAS intervention. Validation through large-scale, prospective multicenter studies is therefore warranted.

## Figures and Tables

**Table 1:** Sociodemographic and preoperative data of pre-ERAS and ERAS cohorts.

	Pre-ERAS	ERAS	
	N (%), Median (IQR)	N (%), Median (IQR)	*p*-Value
Age	58 (52–71)	59 (47–66)	0.437
Gender			0.703
Female	45 (70.30%)	43 (67.20%)	
Male	19 (29.70%)	21 (32.80%)	
Weight (kg)	72 (62–80)	80 (65–86)	0.066
Height (m)	1.65 (1.60–1.71)	1.67 (1.60–1.75)	0.609
BMI	25.67 (23.22–28.91)	27.39 (24.49–30.39)	0.110
Preoperative TSH (mU/L)	1.10 (0.60–1.60)	1.10 (0.57–1.90)	
Preoperative methimazole or PTU			1.000
No	46 (71.90%)	46 (71.90%)	
Yes	18 (28.10%)	18 (28.10%)	
Preoperative D2 vitamin therapy			0.279
No	53 (82.80%)	48 (75.00%)	
Yes	11 (17.20%)	16 (25.00%)	
Thyroiditis			0.080
No	64 (100.00%)	61 (95.30%)	
Yes	-	3 (4.70%)	
Previous neck surgery			0.080
No	64 (100.00%)	61 (95.30%)	
Yes	-	3 (4.70%)	
Previous thyroid surgery			0.080
No	64 (100.00%)	61 (95.30%)	
Yes	-	3 (4.70%)	
Type of surgery			0.343
Lobectomy	8 (12.50%)	9 (14.10%)	
Radicalization	-	2 (3.10%)	
Total thyroidectomy	56 (87.50%)	53 (82.80%)	
Central neck dissection			1.000
No	61 (95.30%)	61 (95.30%)	
Yes	3 (4.70%)	3 (4.70%)	
Surgical time (min)	120 (100–140)	120 (105–143)	0.943
Diagnosis			0.951
Benign/indeterminate disease	54 (84.38%)	52 (81.25%)	
Malignant disease	10 (15.62%)	12 (18.75%)	

IQR: interquartile range; BMI: body mass index; TSH: thyroid-stimulating hormone; PTU: propylthiouracil.

**Table 2:** Differences between pre-ERAS and ERAS cohorts’ results.

	Pre-ERAS	ERAS	
	N (%), Median (IQR)	N (%), Median (IQR)	*p*-Value
Length of stay (days)	2 (2–3)	2 (2–2)	<0.001
Postoperative 6 h calcemia (mg/dL)	8.70 (8.50–9.10)	9.09 (8.90–9.40)	<0.001
POD 1 calcemia (mg/dL)	8.60 (8.20–8.85)	8.81 (8.40–9.20)	0.008
POD 2 calcemia (mg/dL)	8.50 (8.30–9.00)	8.55 (8.17–9.00)	0.816
Postoperative hypocalcemia			0.719
No	37 (57.80%)	39 (60.90%)	
Yes	27 (42.20%)	25 (39.10%)	
Transient hypoparathyroidism			0.309
No	57 (89.10%)	53 (82.80%)	
Yes	7 (10.90%)	11 (17.20%)	
Oral calcium therapy			0.199
No	37 (57.80%)	44 (68.80%)	
Yes	27 (42.20%)	20 (31.30%)	
Intravenous calcium therapy			0.003
No	56 (87.50%)	64 (100.00%)	
Yes	8 (12.50%)	-	
NRS			
Afternoon after surgery	3 (1–6)	2 (0–5)	0.112
POD 1 morning	2 (0–3)	1 (0–4)	0.904
POD 1 afternoon	1 (0–3)	1 (0–2)	0.797
POD 2 morning	0 (0–3)	1 (0–4)	0.317
POD 2 afternoon	0 (0–1)	0 (0–3)	0.295
NRS > 5 in POD 0			0.011
No	47 (73.40%)	58 (90.60%)	
Yes	17 (26.60%)	6 (9.40%)	
Rescue dose			0.213
No	25 (39.10%)	32 (50.00%)	
Yes	39 (60.90%)	32 (50.00%)	
Rescue dose (number of doses)	1 (0–2)	1 (0–2)	0.254
Postoperative opioid therapy			0.310
No	61 (95.30%)	63 (98.40%)	
Yes	3 (4.70%)	1 (1.60%)	
Cephalalgia			0.848
No	44 (68.75%)	45 (70.30%)	
Yes	20 (31.25%)	20 (29.70%)	
PONV			0.048
No	56 (87.50%)	62 (96.90%)	
Yes	8 (12.50%)	2 (3.10%)	
Antiemetic therapy			0.144
No	58 (90.60%)	62 (96.90%)	
Yes	6 (9.40%)	2 (3.10%)	
Complications			0.590
No	36 (56.30%)	39 (60.90%)	
Yes	28 (43.70%)	25 (39.10%)	
Need for surgical revision			NA
No	64 (100.00%)	64 (100.00%)	
Yes	0 (0.00%)	0 (0.00%)	
Vocal cord palsy			1.000
No	63 (98.40%)	63 (98.40%)	
Yes	1 (1.60%)	1 (1.60%)	
Seroma formation			NA
No	64 (100.00%)	54 (94.70%)	
Yes	-	3 (5.30%)	
SSI			0.315
No	64 (100.00%)	63 (98.40%)	
Yes	-	1 (1.60%)	

IQR: interquartile range; POD: postoperative Day; NRS: Numerical Rating Scale; SSI: surgical site infection; PONV: postoperative nausea and vomiting; NA: Not Available.

## Data Availability

Data are contained within the article.
